# Emergence of Azole Resistance in Aspergillus fumigatus and Spread of a Single Resistance Mechanism

**DOI:** 10.1371/journal.pmed.0050219

**Published:** 2008-11-11

**Authors:** Eveline Snelders, Henrich A. L van der Lee, Judith Kuijpers, Anthonius J. M. M Rijs, János Varga, Robert A Samson, Emilia Mellado, A. Rogier T Donders, Willem J. G Melchers, Paul E Verweij

**Affiliations:** 1 Department of Medical Microbiology, Radboud University Nijmegen Medical Centre, Nijmegen, The Netherlands; 2 Nijmegen Institute for Infectious Diseases, Inflammation and Immunity, Radboud University Nijmegen Medical Centre, Nijmegen, The Netherlands; 3 Centraalbureau voor Schimmelcultures (CBS), Fungal Biodiversity Centre, Utrecht, The Netherlands; 4 Department of Microbiology, Faculty of Science and Informatics, University of Szeged, Hungary; 5 Servicio de Micologia, Centro Nacional de Microbiologia, Instituto de Salud Carlos III, Madrid, Spain; 6 Department of Epidemiology and Biostatistics, Radboud University Nijmegen Medical Centre, Nijmegen, The Netherlands

## Abstract

**Background:**

Resistance to triazoles was recently reported in Aspergillus fumigatus isolates cultured from patients with invasive aspergillosis. The prevalence of azole resistance in A. fumigatus is unknown. We investigated the prevalence and spread of azole resistance using our culture collection that contained A. fumigatus isolates collected between 1994 and 2007.

**Methods and Findings:**

We investigated the prevalence of itraconazole (ITZ) resistance in 1,912 clinical A. fumigatus isolates collected from 1,219 patients in our University Medical Centre over a 14-y period. The spread of resistance was investigated by analyzing 147 A. fumigatus isolates from 101 patients, from 28 other medical centres in The Netherlands and 317 isolates from six other countries. The isolates were characterized using phenotypic and molecular methods. The electronic patient files were used to determine the underlying conditions of the patients and the presence of invasive aspergillosis. ITZ-resistant isolates were found in 32 of 1,219 patients. All cases were observed after 1999 with an annual prevalence of 1.7% to 6%. The ITZ-resistant isolates also showed elevated minimum inhibitory concentrations of voriconazole, ravuconazole, and posaconazole. A substitution of leucine 98 for histidine in the *cyp51A* gene, together with two copies of a 34-bp sequence in tandem in the gene promoter (TR/L98H), was found to be the dominant resistance mechanism. Microsatellite analysis indicated that the ITZ-resistant isolates were genetically distinct but clustered. The ITZ-sensitive isolates were not more likely to be responsible for invasive aspergillosis than the ITZ-resistant isolates. ITZ resistance was found in isolates from 13 patients (12.8%) from nine other medical centres in The Netherlands, of which 69% harboured the TR/L98H substitution, and in six isolates originating from four other countries.

**Conclusions:**

Azole resistance has emerged in A. fumigatus and might be more prevalent than currently acknowledged. The presence of a dominant resistance mechanism in clinical isolates suggests that isolates with this mechanism are spreading in our environment.

## Editors' Summary

**Background.:**

Aspergillosis is a group of lung diseases caused by infection with *Aspergillus*, a mold (fungus) that grows on decaying plant matter. Because *Aspergillus* is widespread in the environment, people often breathe in its spores. For most people, this is not a problem—their immune system rapidly kills the fungal spores. However, people with asthma or cystic fibrosis sometimes develop allergic bronchopulmonary aspergillosis, a condition in which the spores trigger an allergic reaction in the lungs that causes coughing, wheezing. and breathlessness. Other people can develop an aspergilloma—a fungus ball that grows in cavities in the lung caused by other illnesses such as tuberculosis. However, the most serious form of aspergillosis is invasive aspergillosis. This pneumonia-like infection, which is fatal if left untreated, affects people who have a weakened immune system (for example, people with leukemia) and can spread from the lungs into the heart, brain, and other parts of the body. Aspergillosis is usually treated with triazole drugs, which inhibit an enzyme that the fungus needs to make its cell membranes; this enzyme is encoded by a gene called *cyp51A*. Voriconazole is the first-line therapy for aspergillosis but itraconazole and posaconazole are also sometimes used and ravuconazole is in clinical development.

**Why Was This Study Done?:**

About half of patients with invasive aspergillosis recover if they are given triazoles. Worryingly, however, strains of Aspergillus fumigatus (the type of *Aspergillus* usually involved in invasive aspergillosis) with resistance to several triazoles have recently been isolated from some patients in The Netherlands. If multi-azole resistant strains of A. fumigatus become common, they could have a serious impact on the management of invasive aspergillosis. However, noone knows what proportion of A. fumigatus strains isolated from patients with aspergillosis are resistant to several azole drugs. That is, noone knows the “prevalence” of multi-azole resistance. In this study, the researchers investigate the prevalence and development of azole resistance in A. fumigatus.

**What Did the Researchers Do and Find?:**

Since 1994, all fungal isolates from patients at the Radboud University Nijmegen Medical Center in the Netherlands have been stored. The researchers' search of this collection yielded 1,908 A. fumigatus isolates that had been collected from 1,219 patients over a 14-year period. Of these, the isolates from 32 patients grew in the presence of itraconazole. All the itraconazole-resistant isolates (which also had increased resistance to voriconazole, ravuconazole, and posaconazole) were collected after 1999; the annual prevalence of itraconazole-resistant isolates ranged from 1.7% to 6%. The researchers then sequenced the *cyp51A* gene in each resistant isolate. Thirty had a genetic alteration represented as TR/L98H. This “dominant resistance mechanism” consisted of a single amino acid change in the *cyp51A* gene and an alteration in the gene's promoter region (the region that controls how much protein is made from a gene). The researchers also analyzed A. fumigatus isolates from patients admitted to 28 other hospitals in the Netherlands. Itraconazole resistance was present in isolates from 13 patients (out of 101 patients) from nine hospitals; the TR/L98H genetic alteration was present in 69% of the itraconazole-resistant isolates. Finally, itraconazole resistance was present in six isolates from four other countries (out of 317 isolates from six countries); only one Norwegian isolate had the TR/L98H genetic alteration.

**What Do These Findings Mean?:**

These findings indicate that azole resistance is emerging in A. fumigatus and may already be more prevalent than generally thought. Given the dominance of the TR/L98H genetic alteration in the azole-resistant clinical isolates, the researchers suggest that A. fumigatus isolates harboring this alteration might be present and spreading in the environment rather than being selected for during azole treatment of patients. Why azole resistance should develop in A. fumigatus in the environment is unclear but might be caused by the use of azole-containing fungicides. Further studies are now urgently needed to find out if this is the case, to measure the international prevalence and spread of A. fumigatus isolates harboring the TR/L98H genetic alteration, and, most importantly, to develop alternative treatments for patients with azole-resistant aspergillosis.

**Additional Information.:**

Please access these Web sites via the online version of this summary at http://dx.doi.org/10.1371/journal.pmed.0050219.

- The MedlinePlus Medical Encyclopedia has a page on aspergillosis (in English and Spanish)
- The UK National Health Service Direct health encyclopedia has detailed information about all aspects of aspergillosis
- The US Centers for Disease Control and Prevention also has information about aspergillosis

## Abstract 3

Paul Verweij and colleagues show that azole resistance has emerged in*Aspergillus fumigatus* in The Netherlands and that a dominant resistance mechanism is present in clinical isolates.

## Introduction

The saprophytic mould *Aspergillus* is known to cause a spectrum of diseases in humans including allergic syndromes, noninvasive infections, as well as invasive aspergillosis. The morbidity and mortality associated with invasive aspergillosis is high, although the mortality rate appears to be improving partly because of the use of effective antifungal agents, most notably the triazoles [1]. Voriconazole was superior to amphotericin B for the treatment of invasive aspergillosis and has become the primary choice to treat this condition [2]. Recently, another triazole, posaconazole, was reported to be highly effective in preventing invasive aspergillosis in neutropaenic patients with acute myeloid leukaemia and myelodysplastic syndrome and in patients with graft-versus-host-disease [3,4]. As a consequence, the role of the class of azole compounds will further increase in the management of invasive aspergillosis.

We recently observed the emergence of multiple-triazole-resistance (MTR) in A. fumigatus, the primary cause of invasive aspergillosis [5]. Patients with MTR aspergillosis presented with primary invasive aspergillosis or with breakthrough infection while receiving itraconazole (ITZ) or voriconazole. The patients were from six different hospitals in The Netherlands, and there was no evidence for spread of a single clone [5]. The isolates were highly resistant to ITZ and showed elevated minimum inhibitory concentrations (MICs) of voriconazole, posaconazole, and the experimental azole ravuconazole [5,6].

The emergence of MTR in A. fumigatus might have significant impact on the role of azoles in the management of invasive aspergillosis, but the impact of resistance on the role of azoles will greatly depend on the prevalence of MTR and the ability to detect MTR isolates at diagnosis [7]. However, the prevalence of MTR is currently unknown, although the number of patients with a MTR isolate was found to be significantly higher than in a previously conducted surveillance study in The Netherlands [5,8]. We investigated the prevalence of azole resistance in A. fumigatus in a prospectively collected fungus culture collection over a period of 14 y. We also investigated the spread of resistance by analysing isolates in our collection, obtained from other hospitals in The Netherlands and other countries.

## Methods

### Setting and Strain Selection

The Radboud University Nijmegen Medical Centre is a tertiary care hospital with beds for 950 patients. The medical microbiology laboratory receives specimens from patients admitted to the hospital as well as those seen as outpatients. The mycology unit of the laboratory has the policy to routinely store fungal isolates from patients with fungal diseases admitted to the hospital or seen as outpatients. For *Aspergillus* species every isolate, irrespective of its clinical significance, was stored. In addition, fungi that were cultured from the hospital environment or those sent to the laboratory for identification, in vitro susceptibility testing or as part of research collaboration, were stored. From the isolates a spore suspension was made in glycerol 10% and stored at −80 °C.

The fungus culture collection was searched for A. fumigatus isolates. To investigate the prevalence of azole resistance we used the A. fumigatus isolates that were cultured from patients that had been admitted to our hospital or were seen as outpatients. The spread of azole resistance was investigated by analysing isolates that had been cultured at other hospitals in The Netherlands and sent to our laboratory for identification. The isolates in our collection obtained from other countries were also screened for azole resistance.

All A. fumigatus isolates were originally identified by experienced technicians on the basis of macroscopic colony morphology, microscopic morphology of conidia and conidia-forming structures, and the ability to grow at 48 °C. The selected A. fumigatus isolates were cultured on Sabouraud slants for 7 d and subsequently subcultured on Sabouraud slants supplemented with itraconazole at a concentration of 8 mg/l, and incubated at 35 °C.

### Phenotypic Analyses

For phenotypic comparisons, for every patient from our hospital with an A. fumigatus isolate that grew in the presence of 8 mg/l of itraconazole (ITZ+), two control patients were randomly selected with an isolate that failed to grow on the itraconazole containing slants (ITZ−): one in the 4 wk preceding the ITZ+ A. fumigatus culture date and one in the 4 wk after the culture date. In addition, one control patient was randomly selected for every remaining month for each year in which a patient with an ITZ+ isolate was identified.

All ITZ+ and ITZ− A. fumigatus control isolates were investigated for their antifungal susceptibility of ITZ, voriconazole, ravuconazole, posaconazole, amphotericin B, terbinafine, and caspofungin using the CLSI M38-A broth micro-dilution reference method [9].

### Genotypic Analyses

For genotypic comparisons, isolates were randomly selected from the ITZ− control patients in a 1:1 ratio with the ITZ+ patients. For ITZ+ and ITZ− isolates the full coding sequence of both strands of the *cyp51A* gene was determined by PCR amplification and analysed to detect mutations [6,10]. Molecular identification was performed by sequencing of parts of the highly conserved β-tubulin and calmodulin gene, as described previously [11–13], in order to rule out any species within the *Aspergillus* section *Fumigati*, which are closely related to A. fumigatus, but are difficult to discriminate based on morphological features alone [12]. The neighbour-joining (NJ) method was used for phylogenetic analysis. Sequences were compared with those of A. fumigatus, A. lentulus, A. viridinutans, *A. brevipes, A. novofumigatus, A. fumigatiaffinis*, and *Neosartorya* species all obtained from the CBS Fungal Biodiversity Centre, Utrecht, The Netherlands [14].

Microsatellite genotyping was used to determine the genetic distances between the ITZ+ and ITZ− A. fumigatus isolates and analysed, as described previously [15,16]. Allele sharing distance matrices were generated [17], and these matrices were used as input to the Neighbor program of the PHYLIP software package to produce the dendrograms [18].

### Patient Characteristics

The electronic patient files were searched for clinical information on the patients with an ITZ-resistant A. fumigatus isolate and from the controls. Information collected included the sample from which the A. fumigatus isolate was cultured, the underlying condition of the patients, and the presence of invasive aspergillosis. Invasive aspergillosis was classified according to the European Organization for Research and Treatment of Cancer and Mycoses Study Group (EORTC)/MSG consensus definitions [19]. Human experimentation guidelines from the Committee on Research Involving Human Subjects Arnhem–Nijmegen were followed in the conduct of this research.

### Statistical Analysis

To test whether ITZ+ isolates had been present throughout the observation period (1994–2007), we calculated the probability that the onset date of finding an ITZ+ was as late as we found it to be. For the phenotypic analysis we used *t*-tests on the natural logarithm of the MIC's and for the genotypic analysis we used a Chi^2^ test. For the patient characteristics, the exact Chi^2^ test was used because of the low expected numbers in certain cells.

## Results

### Prevalence of ITZ+ A. fumigatus Isolates

**Figure 1:**
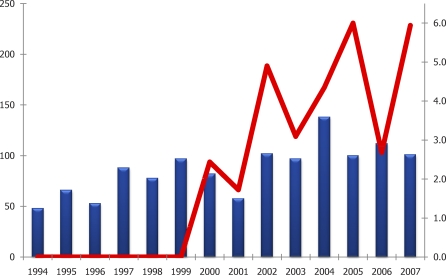
Epidemiology of ITZ Resistance in the A. fumigatus Isolates Blue bars represent the number of patients with a positive A. fumigatus culture (left *y*-axis) and the red line represents the percentage of those patients with an ITZ+ isolate (right *y*-axis). The *x*-axis is the year.

The fungal culture collection included 12,645 isolates that had been collected between 1994 and 2007. Of these, 2,683 were identified as A. fumigatus, and 1,912 were cultured from patients admitted to our hospital or seen as outpatients. These isolates were cultured from 1,219 patients, with a median number of patients of 92.5 per year (range 48 to 138), which corresponded with a median of 130 A. fumigatus isolates per year (range 72 to 215). All isolates were revived and subcultured on Sabouraud slants containing 8 mg/l of ITZ at 35 °C and 1,871 isolates failed to grow under these conditions. However, 41 isolates from 32 patients were able to grow on the ITZ-containing agar. An ITZ+ A. fumigatus isolate was cultured for the first time on June 15, 2000. All 674 consecutive clinical isolates from 462 patients cultured in the 6.5 y before this date did not grow on the ITZ-containing Sabouraud agar. From 2000 onwards, patients were observed every year with an ITZ+ A. fumigatus isolate, with a maximum prevalence of 6.0%. Under the assumption of a random distribution of all ITZ+ isolates over the complete set of *n* observations, the probability of finding the first of *k* occurrences at observation number *o* or later is equal to (*n* − *o* + 1) over *k* divided by *n* over *k*. For the observations at hand this probability is so small (*p* < 0.001), that we can conclude that the ITZ+ isolates are not randomly distributed over all observations: ITZ+ isolates started to occur after a given time point notably after 1994, but before or equal to June 15, 2000 (Figure 1).

### Phenotypic Analysis

**Figure 2:**
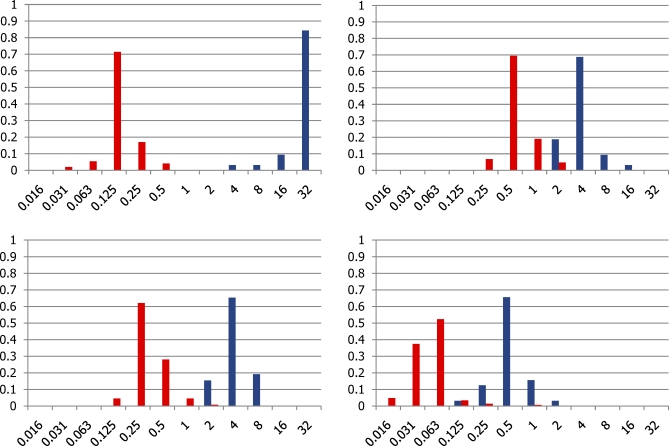
Distribution of Proportions of MICs of 32 ITZ+ A. fumigatus Isolates (Blue Bars) and 115 ITZ− Controls (Red Bars) of ITZ (left upper panel), Voriconazole (right upper panel), Ravuconazole (left lower panel), and Posaconazole (right lower panel) Left *x*-axis represents the proportion of isolates and on the right *x*-axis the MIC distribution is shown.

For phenotypic comparisons, the first ITZ+ isolate of each of the 32 identified patients, was compared with those of the control group. The control group consisted of 115 patients with one ITZ− A. fumigatus isolate per patient, representing at least one patient and isolate from every calendar month between January 1, 2000 and December 31, 2007. The phenotypes of the 32 ITZ+ isolates and the 115 ITZ− control isolates for the azole compounds are shown in Figure 2. All ITZ+ isolates also showed reduced susceptibility to voriconazole, ravuconazole, and posaconazole in comparison with the ITZ− controls (*t*-test, *p* < 0.0005). No significant differences were observed for amphotericin B (*t*-test, *p* = 0.892), terbinafine (*t*-test, *p* = 0.591), and caspofungin (*t*-test, *p* = 0.816) (unpublished data).

### Genotypic Analysis

For genotypic comparisons the first ITZ+ A. fumigatus isolate from 32 patients was compared with those of 32 randomly selected control patients. The dominant genetic changes in the ITZ+ isolates in the *cyp51A* gene were a point mutation at t364a, leading to the substitution of leucine 98 for histidine, together with the presence of two copies of a 34-base pair sequence in tandem in the promoter of *cyp51A* gene (TR/L98H), as previously found [5]. These changes were present in 30 of 32 (94%) ITZ+ isolates and these isolates exhibited the previously reported MTR phenotype. In one ITZ+ isolate an a729g change was found to be responsible for the substitution at codon 220 of methionine by valine (M220V) and in the other no mutations in the *cyp51A* gene were found. In all 32 A. fumigatus ITZ− control isolates, the TR/L98H changes were not observed. To test the relation between an ITZ resistant phenotype and the presence of TR/L98H a Chi^2^ test was performed, and there was a significant relation between the two factors (χ^2^ = 50.143, df = 1, *p* < 0.0001). No changes were found in the *cyp51A* gene of control isolates, except for three isolates, with mutations that were not associated with a resistant phenotype.

**Figure 3:**
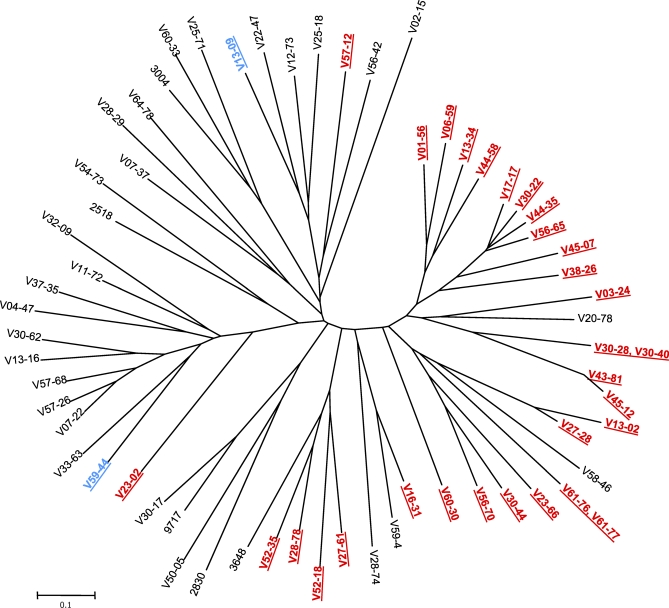
Genotypic Relatedness of 32 ITZ+ A. fumigatus Isolates and 32 ITZ− Controls The numbers are the identification numbers of the individual isolates. The ITZ+ A. fumigatus with the L98H substitution and a tandem repeat in the promoter region of the *cyp51A* gene are underlined and printed in red. The ITZ+ isolates with other or unknown resistance mechanisms are underlined and printed in blue. The ITZ− isolates are printed in black.

The 64 ITZ+ and ITZ− isolates could be classified as A. fumigatus based on sequence analysis of parts of the β-tubulin and calmodulin genes. Three clades were formed based on the neighbour-joining (NJ) analysis of the microsatellite typing; the ITZ+ isolates were genetically different but clustered together in a single clade (Figure 3). Four ITZ+ isolates were clustered within the ITZ− clades, two with the TR/L98H substitution and the others with other or unknown mutations. The branches of the ITZ+ isolates had shorter genetic distances than those within the ITZ− isolate clades.

### Patient Characteristics

In 17 of 32 patients (53%), the ITZ+ isolate was cultured from a respiratory specimen (ten sputum specimens, four bronchoalveolar lavage fluids, and three bronchial secretions), which was not significantly different to 78 of 115 patients (68%) with an ITZ− isolate (41 sputum, 22 bronchoalveolar lavage fluids, and 15 bronchial secretions). A wide variety of underlying diseases and conditions were present (Table 1), most notably patients with chronic lung diseases or cancer. Invasive aspergillosis was diagnosed in nine of 32 patients (28.1%) with an ITZ+ isolate (three proven, three probable, and three possible cases) compared to 25 of 115 (21.7%) in the control group (seven proven, 12 probable, and six possible cases), which was not significantly different. The characteristics of the nine cases with an ITZ+ isolate are shown in Table 2.

**Table 1:**
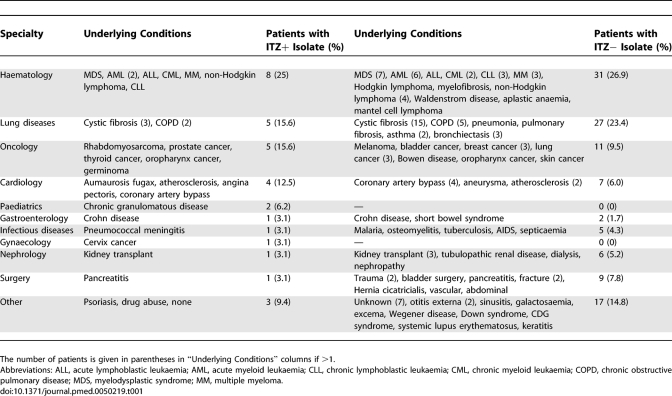
Classification of the Underlying Conditions in 32 Patients with an ITZ+ A. fumigatus Isolate and 115 ITZ− Controls

**Table 2:**
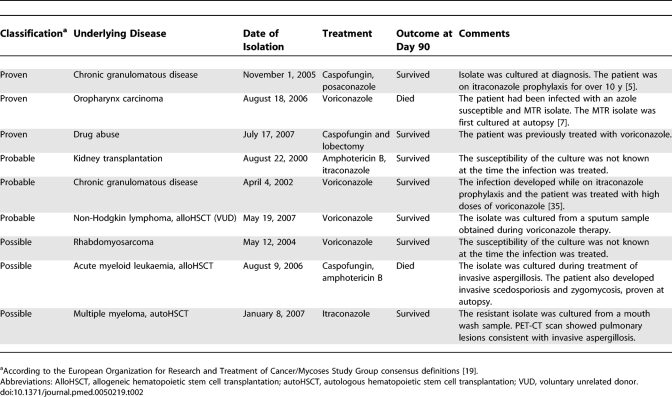
Clinical Characteristics of Nine Patients with Invasive Aspergillosis and an Azole-Resistant A. fumigatus Isolate

### Spread of Azole Resistance

Our culture collection contained 149 A. fumigatus isolates from 28 other hospitals from 20 different cities in The Netherlands. These isolates had been cultured from 101 patients with aspergillus disease and had been sent to our laboratory between 1994 and 2006. Of these, 22 (14.5%) isolates grew on the ITZ-containing Sabouraud slants. The ITZ+ isolates had been cultured from 13 patients (12.8%) from nine different hospitals from seven cities in The Netherlands (Table 3). The first resistant isolate was cultured in 1998, and all others after 2000. The TR/L98H changes were present in nine of 13 (69%) ITZ+ isolates (Table 3).

**Table 3:**
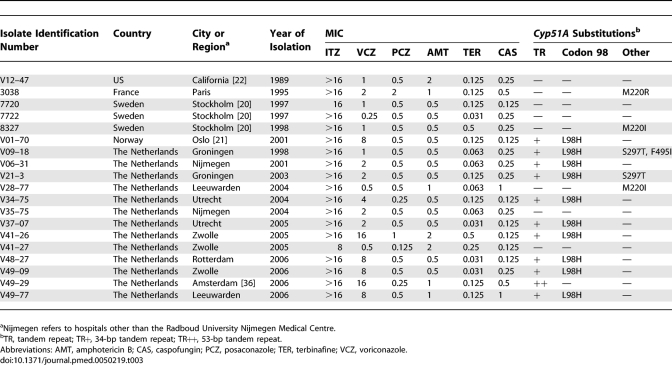
Characteristics of Clinical A. fumigatus Isolates Present in the Fungus Culture Collection Cultured from Other Hospitals in The Netherlands and from Other Countries

Our culture collection contained 317 A. fumigatus isolates from six European countries: Belgium, France, United Kingdom, Sweden, Norway, and Greece. These isolates had been cultured between 1994 and 2002 as part of research collaborations. Six isolates were resistant to itraconazole, originating from France, Sweden, Norway, and the United Kingdom, some of which have been reported previously (Table 3) [6,20–22]. The isolate obtained from the United Kingdom was originally cultured from a patient in the United States in 1989 [22]. All azole-resistant isolates from this group were cultured before 2000, except for the isolate from Norway, which was the only isolate with TR/L98H (Table 3).

## Discussion

Emergence of azole resistance was observed in all three sets of A. fumigatus isolates. Besides our hospital, azole-resistant isolates were found in nine other hospitals in The Netherlands, and in four other countries. The prospectively collected isolates from our hospital indicated that resistance had emerged since 2000 with a prevalence between 1.7% and 6%. Therefore, azole resistance in A. fumigatus is not uncommon and probably more widespread than currently acknowledged.

Recently, taxonomic changes have been proposed for *Aspergillus* section *Fumigati*, with the description of several new species that are closely related to A. fumigatus [12]. Since some of these new species appear to be more resistant to antifungal compounds than A. fumigatus [23], and species demarcation by morphologic features alone is not reliably possible [24], we took great care to correctly identify the species. On the basis of sequence-based analysis, we can be confident that the observed azole resistance has emerged in A. fumigatus.

Acquired resistance against azoles develops in response to exposure of fungi to azole compounds. Azole resistance has been shown to develop following exposure in the patient and in agricultural settings [25–28]. Favourable conditions for the development of azole resistance include long duration of drug exposure and high numbers of reproducing microorganisms [29]. In patients with aspergillus disease, these conditions are present primarily in those with cavities, such as aspergilloma, and indeed numerous resistance mechanisms have been described in isolates obtained from this patient group [25–27]. Among those, changes at codon 220 have been identified, which were also found in isolates in our study. However, isolates that develop azole resistance during patient therapy are unlikely to cause further transmission.

Person-to-person transmission in aspergillus disease is very uncommon, reported only through direct donor-to-recipient contact [30] and through infected wounds [31]. Generally, a patient who responds to therapy overcomes the infection and transmission of the infecting isolate is prevented. The alternative outcome is that a patient fails to respond to therapy, which commonly results in death. Transmission of any isolate after death appears to be unlikely. Acquisition of azole resistance in patients, therefore, is characterized by a high variety of resistance mechanisms and lack of spread.

However, the pattern that we observed in the collection of isolates from our hospital was quite different, with the dominance of the TR/L98H resistance mechanism in clinical isolates from epidemiologically unrelated patients. Furthermore, microsatellite typing showed that the genetic distances between the TR/L98H isolates was shorter than for other azole-susceptible and azole-resistant isolates. These observations suggest that the TR/L98H isolate is not transmitted from person to person, but might be present in our environment. The presence of *Aspergillus* species in the environment is considered the most important risk factor for invasive aspergillosis, and if TR/L98H is present in our environment resistant conidia will be dispersed into the ambient air and may cause infection through inhalation. A. fumigatus resistant to medical azoles has been recovered from the environment, indicating that this mode of transmission is feasible [32]. Furthermore, azole-resistant aspergillosis due to TR/L98H was observed in azole-naïve patients, which supports an environmental origin [5].

It remains unclear how azole resistance develops in A. fumigatus in the environment. We hypothesize that resistance in A. fumigatus could be caused by the use of azole fungicides. Fungicides, including triazoles, are widely used for both plant protection and material protection [33]. As A. fumigatus is a saprophytic mould, environmental azole exposure could lead to the development of multiple resistant mutants. The dominance of the TR/L98H substitution is difficult to explain, but could be due to the procedure we used to select azole-resistant isolates, to selective pressures in the environment, or to properties of these isolates that allow better survival and spread in the environment than isolates with other mutations.

We believe that the distribution of resistance mechanisms within the sets of isolates that we investigated, reflects these two modes of resistance development (patient versus environment). Variable resistance mechanisms were dominant in the resistant isolates from other countries, consistent with development of resistance in individual azole-treated patients. The TR/L98H substitution was the dominant change in the isolates cultured from our hospital, which included all A. fumigatus isolates recovered from clinical specimens irrespective of clinical significance, reflecting acquisition from the environment. The isolates sent to us from other Dutch hospital were exclusively from patients with aspergillus diseases and therefore reflect a mixture of the two modes of resistance development.

Our study was limited by the fact that the isolates from other countries were not collected with the intention to study the epidemiology of resistance. An accurate estimate of the spread of resistance therefore could not be obtained, which would require prospective surveillance studies. Another limiting factor was that we were unable to reliably determine clinical factors that might predispose for azole resistant aspergillosis. This was beyond the primary focus of our study and would require a prospective, multi-centre study.

Given the recent and rapid emergence of the TR/L98H resistance mechanism in A. fumigatus, we believe that further studies are urgently warranted. The reasons for development of resistance and their transmission should be investigated by sampling of the environment and, if resistance is found, establishing a link with azole exposure. In addition, international surveillance studies are required to determine the prevalence and spread of TR/L98H in other countries. Our study does indicate that the TR/L98H substitution has spread to other countries as one isolate from Norway possessed the TR/L98H substitution. In addition, TR/L98H isolates have been reported from Spain [6], Belgium [34], France, and the United Kingdom. One possible scenario might be that TR/L98H isolates have arisen from a single source and are subsequently spreading in the environment across countries, which might be the beginning of the global spread in our environment of azole resistance in A. fumigatus.

It is important to explore the clinical factors that might be associated with the acquisition of TR/L98H isolates and associated disease, and to determine the drug of choice for treatment of patients with azole-resistant aspergillosis. Our data indicate that the activity of voriconazole and posaconazole is reduced against the ITZ-resistant isolates compared with the control isolates. While awaiting the results of confirmative international surveillance studies, we believe that clinical microbiology laboratories should determine the activity of azoles at least in those isolates that are cultured from patients that fail to respond to azole therapy.

## Supporting Information

Alternative Language Abstract S1 Translation of the Abstract into German by T. Schülin (25 KB DOC)

Alternative Language Abstract S2 Translation of the Abstract into Dutch by P. Verweij (25 KB DOC)

Alternative Language Abstract S3 Translation of the Abstract into Spanish by E. Mellado (24 KB DOC)

## Abbreviations

ITZ: itraconazole
MIC: minimum inhibitory concentration
MTR: multiple-triazole-resistance
