# Supplementary material for: Emergence of Azole Resistance in Aspergillus fumigatus and Spread of a Single Resistance Mechanism
Source: PLoS Med. 2008 Nov 11;5(11):e219. doi: 10.1371/journal.pmed.0050219 (PMC2581623; doi:10.1371/journal.pmed.0050219)
Supplement: Supplementary file 1 [file pmed.0050219.sd001.doc]

**Abstract**

**Titel.** Zunahme von Azol-Resistenz von Aspergillus fumigatus und Verbreitung eines einzigen Resistenzmechanismus

**Hintergrund.** Triazol Resistenz wurde von einem Aspergillus fumigatus Isolat eines Patienten mit invasiver Aspergillose beschrieben. Die Prävalenz von Azol-Resistenz von A. fumigatus Stämmen ist nicht bekannt.

**Material, Methoden und Resultate.** Wir untersuchten die Prävalenz von Itraconazol Resistenz von 1908 klinischen A. fumigatus Isolaten der letzten 14 Jahre von 1219 Patienten unseres Universitätsklinikums. Die Verbreitung der Resistenz wurde durch Analyse von 147 A. fumigatus Isolaten von 101 Patienten aus 28 anderen medizinischen Zentren in den Niederlanden und 317 Isolaten aus anderen Ländern ermittelt. Die Isolate wurden mit phänotypischen und molekularen Methoden charakterisiert. Elektronische Patientenakten wurden gebraucht, um Grunderkrankung und Anwesenheit von inversiver Aspergillose festzustellen. 32 von 1219 Patienten hatten ein Itraconazol resistentes Isolat.Alle Fälle wurden nach 1999 gefunden, was einer jährlichen Prävalenz von 1.7% – 6% entspricht. Die Itraconazol resistenten Isolate hatten auch eine erhöthe MHK von Voriconazol, Ravuconazol und Posaconazol.Als Hauptresistenzmechanismus wurde eine Substitution von Leucin98 statt Histidine im cyp51A Gen gefunden, zusammen mit einer 34bp Tandem-Sequenz im Promoter Gen (TR/L98H)

Eine Microsatteliten Analyse zeigte, dass die Itraconazol resistenten Isolate genetisch unterschiedlich waren, aber lokal gehäuft vorkamen. Itraconazol empfindliche Isolate waren nicht häufiger Ursache einer invasiven Aspergillose als Itraconazol resistente Isolate. Isolate von 13 Patienten (12,8%) aus 9 medizinischen Zentren in den Niederlanden waren Itraconazol resistent, 69% diese Isolate besassen die TR/l98H Substiution. Weitere 6 dieser Isolate kamen aus anderen Ländern.

**Fazit.** Azol resistenz in A. fumigatus hat zugenommen und ist wahrscheinlich prävalenter als angenommen. Das Vorkommen von Resistenzmechanismen in klinischen Isolaten lässt vermuten, dass dieser Mechanismus sich in der Umwelt verbreiten könnte.
