# Supplementary material for: Emergence of Azole Resistance in Aspergillus fumigatus and Spread of a Single Resistance Mechanism
Source: PLoS Med. 2008 Nov 11;5(11):e219. doi: 10.1371/journal.pmed.0050219 (PMC2581623; doi:10.1371/journal.pmed.0050219)
Supplement: Supplementary file 2 [file pmed.0050219.sd002.doc]

**Samenvatting**

**Titel.** Het verschijnen van azool-resistentie in *Aspergillus fumigatus* en de verspreiding van één dominante resistentie mechanisme.

**Achtergrond.** Resistentie tegen triazolen werd onlangs gerapporteerd bij *Aspergillus* *fumigatus* isolaten van patiënten met invasieve aspergillose. De prevalentie van azool-resistentie is onbekend.

**Methoden en resultaten.** We hebben 1908 klinische *A. fumigatus* isolaten onderzocht op de aanwezigheid van resistentie tegen itraconazole verzameld van 1219 patiënten in ons Universitair Medisch Centrum over een periode van 14 jaar. De verspreiding van resistentie werd onderzocht in 147 *A. fumigatus* isolaten van 101 patiënten, afkomstig van 28 andere Nederlandse ziekenhuizen, en 317 isolaten uit zes andere Europese landen. De isolaten werden gekarakteriseerd door gebruikmaking van fenotypische en moleculaire methoden. De elektronische patiënten dossiers werden gebruikt om de onderliggende ziekten van de patiënten te onderzoeken en de aanwezigheid van invasieve aspergillose.

Itraconazol resistente isolaten werden gevonden in 32 van de 1219 patiënten. Alle gevallen werden geobserveerd na 1999 met een jaarlijkse prevalentie van 1,7% tot 6%. De itraconazol resistente isolaten toonde eveneens verhoogde minimaal remmende concentraties voor voriconazol, ravuconazol en posaconazol. Een substitutie van leucine 98 voor histidine in de *cyp51A* gen, samen met twee kopieën van een 34 bp sequentie in tandem in de promotor regio van het gen (TR/L98H) werd gevonden als dominante resistentie mechanisme. Microsatelliet genotypering toonde dat de itraconazol resistente stammen genetisch verschillend waren maar wel clusterden. Het aantal gevallen van invasieve aspergillose was vergelijkbaar voor patiënten met een itraconazol resistentie stam en een itraconazol gevoelige stam. Resistentie tegen itraconazol werd gevonden in isolaten van 13 patiënten (12,8%) afkomstig uit 9 andere Nederlandse ziekenhuizen, waarbij 69% de TR/L98H resistentie aanwezig was, en in zes stammen afkomstig uit vier andere landen.

**Conclusies:** Azool-resistentie is verschenen in *A. fumigatus* en komt vaker voor dan momenteel wordt erkend. De aanwezigheid van een dominante resistentie mechanisme suggereert dat dit mechanisme zich aan het verspreiden is in onze omgeving.
