# Supplementary material for: Emergence of Azole Resistance in Aspergillus fumigatus and Spread of a Single Resistance Mechanism
Source: PLoS Med. 2008 Nov 11;5(11):e219. doi: 10.1371/journal.pmed.0050219 (PMC2581623; doi:10.1371/journal.pmed.0050219)
Supplement: Supplementary file 3 [file pmed.0050219.sd003.doc]

**Resumen:**

**Título.** Aparición y diseminación de cepas de *Aspergillus fumigatus* resistentes a los azoles con un mismo mecanismo de resistencia.

**Antecedentes.** Recientemente se ha comunicado la aparición de cepas de *Aspergillus fumigatus*, resistentes a triazoles, aisladas de pacientes con aspergillosis invasora. En la actualidad se desconoce la prevalencia de la resistencia a azoles en *A. fumigatus.*

**Métodos y Resultados.** Hemos estudiado la prevalencia de la resistencia a itraconazol (ITZ) en 1.908 cepas clínicas de *A. fumigatus* procedentes de 1.219 pacientes de nuestro Centro Médico Universitario durante un periodo de 14 años. El estudio de la diseminación de cepas resistentes se realizó analizando 147 cepas *de A. fumigatus* obtenidas de 101 pacientes procedentes de 28 centros médicos en Holanda y 317 cepas de otros seis países. Las cepas se identificaron en base a sus características fenotípicas y se tipificaron utilizando métodos moleculares. El diagnóstico de aspergilosis invasora, así como los datos clínicos de los pacientes, se obtuvieron de los archivos de sus historias clínicas.

En total se encontraron 32 cepas resistentes a itraconazol entre los 1,219 pacientes. Todos los casos fueron posteriores al año 1999, con una prevalencia anual desde el 1.7% al 6%. Las cepas resistentes a ITZ también presentaban elevación de las concentraciones mínimas inhibitorias a voriconazol, ravuconazol y posaconazol. El mecanismo de resistencia que se encontró con mayor frecuencia fue la substitución de leucina 98 por histidina en el gen cyp51A acompañado siempre de una repetición en tándem de una secuencia de 34 pb en el promotor del mismo gen (TR/L98H).

El análisis de microsatélites indicó que las cepas resistentes no eran clónales si bien se encontraban genéticamente agrupadas en un cluster. No se encontraron diferencias de aislamiento de cepas sensibles o resistentes a itraconazol de pacientes con aspergilosis invasora. Se aislaron cepas resistentes a ITC en 13 pacientes (12.8%) pertenecientes a otros 9 centros médicos en Holanda, de los cuales 69% presentaban el mecanismo de resistencia TR/L98H, y en seis cepas procedentes de otros cuatro países.

**Conclusiones:** Se confirma la aparición de resistencia a los azoles en *A. fumigatus* y se alerta que puede ser mas frecuente de lo que actualmente se piensa. La presencia de un mismo mecanismo de resistencia en la mayoría de las cepas sugiere la diseminación de estas cepas resistentes en el medio ambiente.
